# Harmonizing the past: EEG-based brain network unveil modality-specific mechanisms of nostalgia

**DOI:** 10.3389/fpsyg.2025.1517449

**Published:** 2025-01-22

**Authors:** Hu Shuxiang, Liu Ying, Yue Qizong, Zhao Huan, Zheng Maoping

**Affiliations:** ^1^School of Music, Southwest University, Chongqing, China; ^2^School of Music, China Music Mental Health Institute, Southwest University, Chongqing, China

**Keywords:** nostalgia, audiovisual channel, emotional intervention, brain network, nostalgic music

## Abstract

**Introduction:**

Nostalgia is a complex emotional experience involving fond memories of the past and mild sadness, characterized by positive emotions associated with reflecting on previous events. It can awaken emotional memories of loved ones or significant events, contributing to an increase in positive emotions. An unresolved question regarding nostalgia is whether different channels of nostalgia input exhibit distinct mechanisms.

**Methods:**

This study examined the emotional and neural effects of nostalgia using various sensory channels through behavioral experiments and electroencephalography (EEG) measurements conducted with college students in China. Participants' emotions were elicited using nostalgic and non-nostalgic stimuli presented through three different sensory channels: auditory (sound only), visual (e.g., still images or synchronized lyrics related to music), and audiovisual (a combination of sound and visual elements, such as music videos).

**Results:**

The results demonstrated that nostalgic stimuli elicited significantly higher levels of emotional arousal, pleasure, nostalgia, and dominance compared to non-nostalgic stimuli. At the neural level, nostalgic stimuli enhanced the connection strength, global and local efficiency, and diminished eigenpath length of brain networks in the alpha and gamma bands. Additionally, nostalgia through the auditory channel induced higher activity intensity in the theta and gamma bands and increased brainwave amplitudes in the alpha bands. The audiovisual channel was capable of triggering stronger alpha-wave responses than the visual channel alone.

**Discussion:**

These findings suggest that nostalgia effectively triggers positive emotional states and enhances cognitive processing. The audiovisual channel, in particular, showed advantages in eliciting alpha-wave responses. Further research is needed to explore the potential of nostalgia as an adjunctive therapeutic tool.

## 1 Introduction

Nostalgia is a complex emotional state characterized by a longing and attachment for past experiences (Batcho, 1995). While it can evoke feelings of sadness and yearning (Barrett et al., 2010; Mills and Coleman, 1994), it is also recognized as a valuable emotional resource that positively impacts mood and well-being. It is a ubiquitous experience, with research indicating that 96% of people feel nostalgic at least once a month and 79% at least once a week (Wildschut et al., 2006). In today's rapidly changing world—accelerated by globalization and technological advances—traditional cultures and community structures have transformed dramatically. People increasingly face identity crises and diminished collective belonging (Stephan et al., 2015). Nostalgia has thus become a coping mechanism for individuals to deal with social upheaval and temporarily escape present anxieties by reminiscing about the past.

When people feel loneliness, anxiety, or helplessness, they often experience nostalgia (Routledge et al., 2013). Nostalgia in such situations can alleviate negative emotions, enhance self-identity, and improve psychological resilience (Routledge et al., 2013; Webster, 1993). Moreover, nostalgia fosters social cohesion and emotional connections, as individuals find psychological solace through shared memories and cultural identity. This, in turn, fosters a sense of solidarity during difficult times (Abakoumkin and Green, 2021).

Sensory inputs like music and images are potent in eliciting nostalgia (Newman et al., 2020). Music, in particular, is strongly tied to key moments in life. Listening to old favorite songs can evoke pleasant memories and strengthen social bonds (Janata, 2009). Studies that use music to evoke nostalgia further indicate that familiar, autobiographically salient, and emotionally positive musical pieces increase activation in regions associated with autobiographical memory and self-referential processing (Barrett et al., 2010; Janata, 2009; Trost et al., 2012; Yang et al., 2023). For example, Janata (2009) found that such compositions elicit responses in the mPFC (medial Prefrontal Cortex), hippocampus, and PCC (Posterior Cingulate Cortex), supporting the role of these regions in processing personally meaningful memories triggered by music. These neural correlates underscore the significance of music as a potent cue for nostalgic experiences.

Although neuroscientists have explored various modalities that induce nostalgia—such as auditory (Barrett et al., 2010; Ford and Merchant, 2010; Janata, 2009; Trost et al., 2012), visual (Oba et al., 2016; Speer et al., 2014), and olfactory (Matsunaga et al., 2013) cues—the effects of audiovisual integration on nostalgic experiences remain understudied. Multisensory integration, in particular the combination of auditory and visual channels, is a complex neural process crucial to various cognitive and social functions (Gelder and Bertelson, 2003; Lian et al., 2023; Ruffman et al., 2009). The simultaneous presentation of auditory and visual stimuli can enhance information processing efficiency, emotional intensity, and the speed of emotional recognition (Föcker et al., 2011; Müller et al., 2012). Research shows that emotionally congruent audiovisual inputs (e.g., upbeat music paired with happy images) intensify and prolong emotional experiences, while incongruent inputs (e.g., sad music with happy images) disrupt emotional coherence and require additional cognitive resources for resolution (Gao et al., 2020; Pan et al., 2019). These findings indicate that multisensory channels interactions can amplify or attenuate emotional responses, depending on stimulus congruence.

Despite these insights into how audiovisual integration affects emotion, the study of how multisensory inputs influence nostalgia remains scarce. As such, the present research aims to provide a systematic examination of how audiovisual integration influences both the subjective intensity of nostalgia emotions and the underlying neural activity. To address these issues, the current study explores how nostalgia presented via different sensory channels affects emotional responses and associated neural activations. Specifically, we examine whether combining auditory and visual nostalgic stimuli evokes more intense nostalgic feelings and pronounced neural responses compared to auditory- or visual-only presentations.

## 2 Methods

### 2.1 Participants

We invited 38 first-year college students (18 men, 20 women; mean age = 18.79 ± 1.01 years) from different departments at a university in Zhengzhou City, China as the participants through campus announcements or via the online platform Sojump. Music majors and students with music learning experience were excluded to avoid the influence of music learning and training experience on the experimental results. All participants had normal hearing and speech, and were right-handed with corrected or uncorrected normal vision. The participants had no mental health issues or history of brain injury. Participants were instructed to avoid stressful or strenuous activities leading up to the experiment to ensure relaxation and optimal performance.

To ensure the validity of the experiment, all participants were asked to avoid staying up late or being overworked the night before the experiment, to ensure sufficient rest, and to avoid consuming food or beverages containing caffeine. Additionally, participants were instructed to avoid alcohol consumption prior to the experiment. Participation was voluntary, and participants were fully informed about the purpose, procedures, and potential risks, providing written informed consent prior to the experiment. Participants could withdraw from the study at any time. The study adhered to ethical guidelines such as the Declaration of Helsinki, privacy and confidentiality were rigorously maintained. The participants received a compensation of 40–60 RMB for their time and participation after completing our experiment. This study was approved by the Ethics Committee of Ethics Committee of Southwest University (approval number H24188).

### 2.2 Experimental materials

As nostalgic music is usually closely associated with autobiographical memories, differences exist in preferences for nostalgic music among individuals of different ages. Based on the previous studies, we selected nostalgic music from the time period, in which the autobiographical memories were most concentrated. We used the following sources for nostalgic music: (1) music popular between 1999 and 2022 was chosen to align with the childhood and teenage years of the university students, as these are formative phases for developing music preferences and nostalgic connections. By selecting tracks from this era, we aimed to include songs likely to evoke the vivid personal memories and emotional resonance, making them well-suited for studying the influence of nostalgia on emotional responses; (2) nostalgia-inducing materials for college students used in previous studies in China; and (3) nostalgic music perceived by 20 freshmen based on interviews. Participants were asked to recall songs that evoked nostalgic feelings and describe their personal connections to these tracks. The interviews followed a semi-structured format, allowing for in-depth exploration of their memories and emotions associated with the music. Responses were then analyzed to determine common themes and specific songs that were perceived as nostalgic, ensuring a comprehensive understanding of the participants' experiences. We selected 60 pieces of music classified into five categories: children's songs and nursery rhymes (e.g., Let's Swing the Oars, The Big Windmill, and Big Ears Tutu), game songs (e.g., Against the Odds and Sailor Moon), songs glorifying the motherland (e.g., My Motherland and Dongfanghong), popular songs (e.g., Porcelain with Blue Flowers, Those Were the Years, and Little Apple), and traditional operas (e.g., Lady General Mu Takes Command and Who Says Women Are Not as Good as Men). In addition, five songs that were familiar to college students but did not easily trigger nostalgia were selected as control group materials. These songs were primarily popular tracks that had been recently released during the experimental cycle. The selection process involved surveying college students to identify songs they recognized but reported minimal nostalgic associations, ensuring that these songs effectively served as neutral stimuli in comparison to nostalgic music.

Subsequently, we conducted an online questionnaire survey, which was divided into two parts: (1) basic information and personal music preferences and (2) a matrix scale in which the participants were asked to rate music videos. The rating dimensions were familiarity (1 = very unfamiliar; 5 = very familiar), nostalgia (1 = very un-nostalgic; 5 = very nostalgic), and pleasantness (1 = very unpleasant; 5 = very pleasant). Each audio and video clip was played for 30–40 s. We collected 322 questionnaires, of which 257 were valid with an effective recovery rate of 79.8%. Based on the familiarity, nostalgia, and pleasure scores, 30 pieces of music with the strongest sense of nostalgia, highest familiarity, and strongest sense of pleasure were selected as experimental materials (Supplementary Table S1). Non-nostalgic music was selected based on two criteria: First, contemporary music (e.g., released within the past 3 years) was chosen that the participants enjoyed casually without part of their lives long enough to develop the nostalgic associations. Second, the selection included the instrumental tracks or songs with simplistic or non-specific lyrics, which are less likely to evoke the personal reflection. Ultimately, 30 tracks were selected as the auditory materials for the formal experiment, ensuring the effective controls in the study of nostalgia.

Nostalgic visual materials were primarily sourced from a nostalgia-themed image library, featuring images with high nostalgic value, including games, objects, and activities. In contrast, non-nostalgic visual materials were obtained from the Geneva Affective Picture Database, comprising neutral images. This selection process ensured a clear distinction between nostalgic and non-nostalgic visual stimuli for the study.

Nostalgic audiovisual materials primarily consist of music videos, derived from the selected songs, along with opening sequences from animated series or television shows. Non-nostalgic audiovisual materials are composed of neutral videos, which are created by combining non-nostalgic visual and auditory components edited together using video software.

### 2.3 Experimental design

The current study employed a 3 (channel type: visual, auditory, audiovisual) × 2 (music type: nostalgic, non-nostalgic) design. Both Channel type and music type were within-subjects variables.

### 2.4 Experimental procedures

This study used a passive listening paradigm by combining subjective reports with electroencephalography (EEG) measurements. Each participant has to complete 36 trials (six trial for practice section + 30 trial for normal experiment). A practice section was conducted prior to formal experiment for all participants to familiarize them with the experiment procedures. The data from practice sections were not included in the analysis of the experimental data.

**Figure 1:**
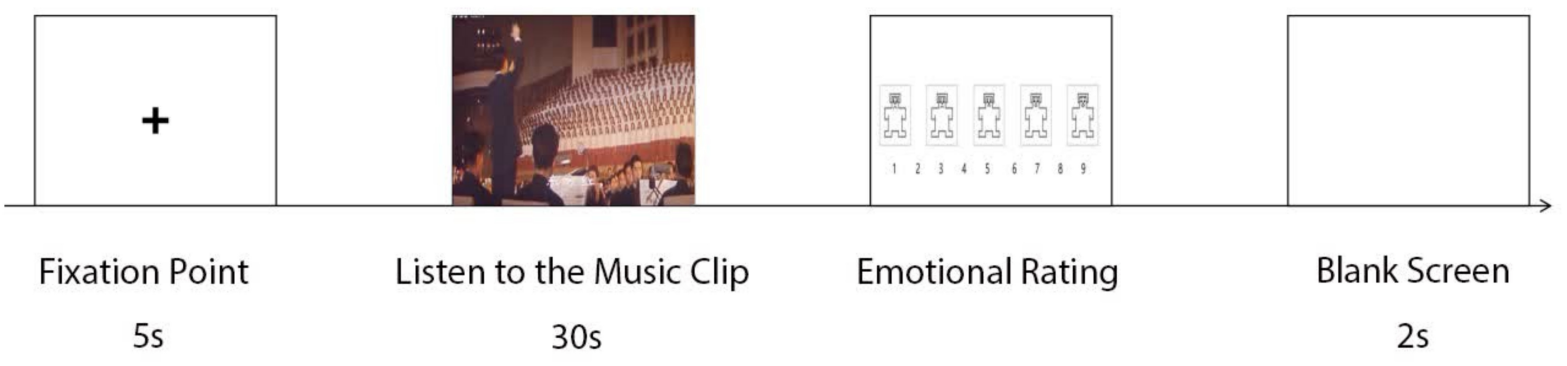
Experimental flow.

In each trial, participants first saw a gaze point on a gray background screen for 5 s. Subsequently, a piece of audio or visual or audiovisual material presented for 30 s. After the presentation of the material, the participants reported their state nostalgia and emotion responses, including pleasantness, arousal, and dominance with a nine-point Likert scale (1 = very low; 9 = very high). After trial completion, the screen displayed a 3-s blank interval and then proceeded to the next trial (Figure 1).

Participants reported their state nostalgia with the three-question State Nostalgia Scale (Wildschut et al., 2006), which was designed to evaluate participants' momentary nostalgic experiences. Responses to the questions are recorded on a nine-point Likert scale (1 = Strongly Disagree, 9 = Strongly Agree), and the average scores were calculated to reflect the overall intensity of state nostalgia. The scale demonstrates high internal consistency (e.g., Cronbach's alpha > 0.9) and is widely applied in psychological researches to examine the immediate emotional and cognitive effects of nostalgia. After the nostalgia scale, participants' emotional responses were measured using the Self-Emotion Rating Scale (Bradley and Lang, 1994), which included three questions as well, evaluating dimensions of their experienced pleasure, arousal, and dominance. Moreover, the duration between the onset of each scale question and participant's report by a key press, were also recorded.

We also recorded participants' EEG activities using a 64-conductor device (Brain Products, Germany) with a 64-conductor polar cap based on the International 10–20 System Extension. Whole-head averaging was used as a reference during recording to ensure that the impedance between all electrodes and the scalp was < 5 kΩ. In addition, horizontal electrooculograms from the lateral aspects of both eyes and vertical electrooculograms from the upper and lower orbital areas of the left eye were recorded simultaneously.

In the formal experiment, the stimulus materials were arranged in the following sequence: first visual, then auditory and audiovisual in the last. This operation aims to minimize channel-switching effects and the influence of dual-channel audiovisual information on single-channel processing. To avoid interference between channels, stimuli materials were not repeated used for different channels, i.e., all 30 stimuli will appear only once for each participant. Each channel contained 10 trials, half with nostalgic stimuli and half with non-nostalgic stimuli, randomly arranged. After completing a channel, participants were allowed to take a 2-min break to prepare for the next part of the experiment.

### 2.5 Statistical analysis

#### 2.5.1 Behavioral data analysis

Behavioral data, including emotional ratings (pleasure, arousal, and dominance), nostalgic feeling scores, and key press reaction times, were analyzed using SPSS 26.0. Outliers for each indicator, defined as data points exceeding ±3 *SD* from the mean, were excluded to ensure data quality. For the statistical analyses of these indicators, repeated measures Analysis of Variance (ANOVA) was employed to examine the main effects and interaction effects across different experimental conditions. The Greenhouse-Geisser correction was applied to adjust *p*-values for all main effects and interaction effects, ensuring the robustness and reliability of the results.

#### 2.5.2 EEG data analysis

The EEG raw data from two individuals were damaged and unavailable, resulting in a total of 36 EEG samples available for analysis. The EEG data of interest were collected during the presentation of nostalgic stimuli to capture the neural responses elicited by these stimuli. The data were filtered by applying band-pass filtering (0.1–50 Hz), and the effects of industrial frequency noise were effectively removed by adding a notch filter (49–51 Hz). Interpolation techniques were used to address the effects of bad electrodes and fix possible missing electrode signals in the data, considering data quality. Subsequently, the EEG signals were segmented into epochs aligned with the onset of each stimulus to facilitate comparisons across the various experimental conditions. Each epoch encompassed the entire duration of the stimulus presentation, which lasted for ~45 s, thereby capturing sustained neural activity associated with prolonged stimulus exposure. We employed frequency domain analysis and relative power measurements to assess changes in EEG frequency bands across the various experimental conditions. In order to eliminate the interference of the baseline shift in the subsequent analysis, a baseline correction process was performed to adjust the signal to the average level during the baseline period. Finally, the data were processed to remove artifacts such as ocular movements (e.g., eye blinks) and head movements to improve the purity and interpretability of the data using independent component analysis (ICA). Ocular artifacts were corrected by applying ICA, where components corresponding to eye movements were identified and removed based on their characteristic topographies and time courses. The EEG data were then re-referenced from whole-head averaging to linked left and right mastoid references to enhance signal quality. Artifactual signals with amplitudes exceeding ±75 μV were automatically excluded. These preprocessed data provided a basis for scalp topography and functional brain network analyses.

#### 2.5.3 Brain network construction and analysis

In establishing EEG functional brain networks, this study directly uses the EEG channels on the scalp as the nodes of the network. We utilized the Weighted Phase Lag Index (WPLI) as a measure of connection strength between nodes to construct functional brain networks. WPLI is based on the phase differences between EEG signals and calculates the weighted average of the imaginary part of these phase differences, emphasizing connections with consistent phase lag. Compared to the traditional Phase Lag Index (PLI), WPLI effectively reduces the influence of volume conduction and common source interference on synchronization measurements, providing a more accurate reflection of true functional connectivity between different regions of the brain (Vinck et al., 2011).

We first preprocessed the EEG data from 36 participants, extracting signals in the following frequency bands of interest: Delta (δ: 1–4 Hz), Theta (θ: 4–8 Hz), Alpha (α: 8–13 Hz), Beta (β: 13–30 Hz), Gamma (γ: 30–50 Hz). These frequency bands were selected based on their established relevance to cognitive and emotional processing in the literature. Subsequently, we calculated the WPLI values between each pair of channels to generate a functional connectivity matrix, where nodes represent EEG channels and edge weights correspond to the WPLI values. For the complex network analysis, we calculated network metrics to evaluate the topological structure and information transmission efficiency of the networks. The metrics includes Local Properties and Global Properties. The Local Properties contain the Node Strength, which measures the sum of connection strengths for each node, and the Clustering Coefficient, which assesses the degree to which nodes tend to cluster together. The Global Properties contain Characteristic Path Length, which indicates the average shortest path between all pairs of nodes, the Global Efficiency, which reflects the efficiency of information exchange across the entire network, and the Local Efficiency, which measures the efficiency of information exchange within the immediate neighborhood of a node.

Additionally, to assess the intensity of neural oscillatory activity across different nostalgic sensory channels, Power Spectral Density (PSD) was calculated for the acquired EEG time-series signals. PSD quantifies the power within specific frequency bands, providing a measure of the strength of neural oscillations associated with each band. This analysis facilitates the comparison of neural activity intensity across different sensory channels (auditory, visual, and audiovisual) during the listening of nostalgic music, thereby elucidating the differential neural responses elicited by each sensory modality.

## 3 Results

### 3.1 Nostalgia manipulation test

We used paired samples *t*-test to determine the differences in nostalgia produced by nostalgic and non-nostalgic stimuli. In the visual, auditory, and audiovisual channels, nostalgic materials evoked significantly more nostalgic emotions than non-nostalgic ones (Table 1). This indicated that the selected nostalgic materials were able to evoke nostalgia and that the nostalgia manipulation was established.

**Table 1:** Manipulation test for nostalgia.

**Factor**	**Nostalgia (*N* = 38)**	**Non-nostalgia (*N* = 38)**	***t***	***p***
	***M ±SD***	***M ±SD***		
Visual	7.52 ± 0.95	4.16 ± 1.57	3.770	0.000
Auditory	7.34 ± 0.88	3.90 ± 1.81	14.056	0.000
Audiovisual	7.81 ± 0.65	4.13 ± 1.80	23.186	0.000

### 3.2 Effects of nostalgia type and channel type on state nostalgia and emotional responses

#### 3.2.1 Effects on state nostalgia

In order to examine the effects of channel type (auditory, visual, audiovisual) and nostalgia type (nostalgia, non-nostalgia) on state nostalgia, we conducted a 3 × 2 repeated measures ANOVA. The results indicated a significant main effect of channel type, *F*_(2, 74)_ = 3.668, *p* < 0.05, η2 p = 0.090. Participants felt more nostalgic with auditory (*M* = 5.65) than visual (*M* = 5.34), *p* < 0.05, audiovisual (*M* = 5.76) is higher than visual (*M* = 5.34), *p* < 0.001; and audiovisual (*M* = 5.76) is higher than auditory (*M* = 5.65), *p* = 0.56. The main effect of nostalgia type was significant, *F*_(1, 37)_ = 516.835, *p* < 0.001, η2 p = 0.993, with the score of nostalgia (*M* = 7.55) is significant higher than non-nostalgia (*M* = 3.68). More importantly, a significant interaction between channel type and nostalgia type emerged, *F*_(2, 74)_ = 3.483, *p* < 0.05, η2 p = 0.14, suggesting that the influence of channel type on state nostalgia differed depending on the sensory channel through which the stimuli were presented. Simple effect analysis shows that on nostalgic stimuli, the state nostalgia was stronger with audiovisual (*M* = 7.79) than visual (*M* = 7.35), *p* < 0.05, the state nostalgia was stronger with auditory (*M* = 7.65) than visual (*M* = 7.35), *p* < 0.05, audiovisual (*M* = 7.79) is higher than auditory (*M* = 7.65), but not significant, *p* = 0.12. On non-nostalgic stimuli, the state nostalgia was stronger with audiovisual (*M* = 3.8) and auditory (*M* = 3.86) than visual (*M* = 3.29), *p*_*s*_ < 0.05, but the difference between audiovisual (*M* = 3.8) and auditory (*M* = 3.86) was not significant, *p* = 0.84.

#### 3.2.2 Effects on emotional response levels

**Figure 2:**
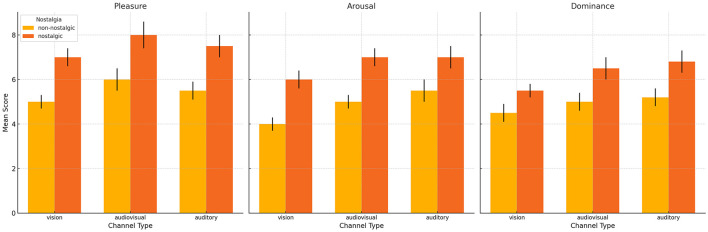
Interactive bar charts—pleasantness, arousal, dominance by music & channel.

In order to examine the effects of channel type (auditory, visual, audiovisual) and nostalgia type (nostalgia, non-nostalgia) on emotional response levels, we conducted 3 × 2 repeated measures ANOVA for pleasure, arousal, and dominance, respectively (Figure 2). Descriptive statistics for emotional response levels are presented in Table 2. The results showed a main effect of nostalgia type on pleasure [*F*_(1, 37)_ = 299.349, *p* < 0.001, η2 p = 0.890], arousal [*F*_(1, 37)_ = 342.762, *p* < 0.001, η2 p = 0.903], and dominance [*F*_(1, 37)_ = 14.723, *p* < 0.001, η2 p = 0.285], respectively, with all the emotional response levels of nostalgic stimuli is significantly higher than that of non-nostalgic stimuli. However, the main effect of channel type and the interaction effect on these emotional responses was not significant.

**Table 2:** Descriptive statistical tables of emotional response levels (*M* ± *SD*).

**Response level**	**Nostalgic stimuli**			**Non-nostalgic stimuli**		
	**Auditory**	**Visual**	**Audiovisual**	**Auditory**	**Visual**	**Audiovisual**
Pleasure	7.132 ± 1.002	6.991 ± 0.84	7.192 ± 0.986	4.916 ± 0.86	4.57 ± 0.928	5.209 ± 0.648
Arousal	7.114 ± 0.94	6.877 ± 1.115	7.209 ± 1.063	4.937 ± 1.069	4.507 ± 0.984	4.798 ± 1.183
Dominance	7.13 ± 1.308	6.84 ± 1.491	7.344 ± 1.301	6.402 ± 1.515	6.0 ± 1.809	6.486 ± 1.616

### 3.3 Brain networks results

**Figure 3:**
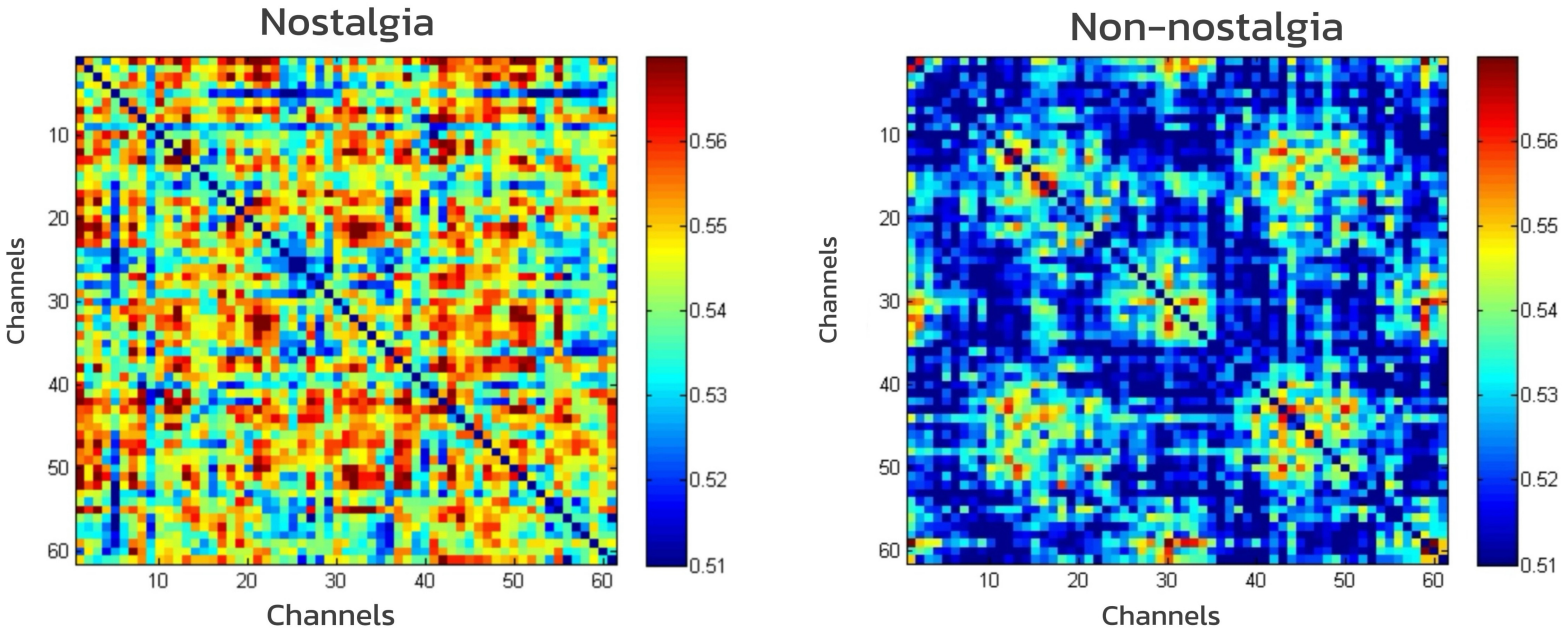
Matrix of the network constructed based on WPLI in alpha band.

**Figure 4:**
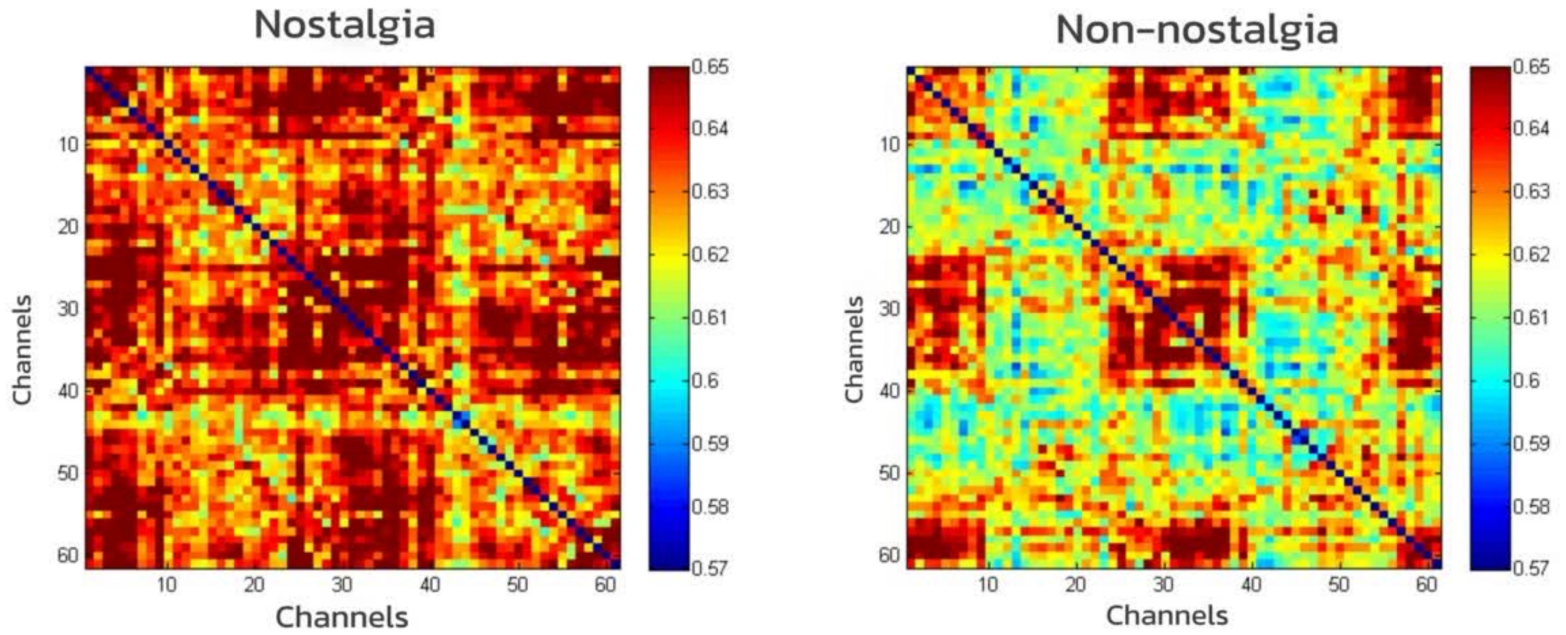
Matrix of the network constructed based on WPLI in gamma band.

For each frequency band, whole-brain functional networks were constructed separately for the nostalgic and non-nostalgic emotional states. Paired-sample *t*-tests were conducted to compare differences in the properties of these whole-brain networks between the different emotional states. The WPLI matrix plot was obtained by calculating the mean of the data and analyzing it using the weighted phase lag index. The results showed that the rhythms of the alpha (*M* = 0.53, *SD* = 0.095 vs. *M* = 0.51, *SD* = 0.092; *t* = 70.82, *p* < 0.001) and gamma bands (*M* = 0.64, *SD* = 0.11 vs. *M* = 0.60, *SD* = 0.12; *t* = 117.73, *p* < 0.001) were significantly higher for nostalgic stimuli compared to non-nostalgic stimuli, indicating that nostalgic stimuli enhanced functional connectivity in the brain (Figures 3, 4).

The analysis of the neural network properties encompassed four metrics: strength, global efficiency, local efficiency, and characteristic path length. The overall effects of the full model (i.e., 3 × 2 ANOVA) on these metrics were not significant. Consequently, we separately examined the effects of nostalgia type and channel type on each metric. The results revealed no significant differences in whole-brain network properties between nostalgic and non-nostalgic stimuli within the beta and theta bands. The connection strength for nostalgic stimuli was significantly higher than that for non-nostalgic stimuli in the alpha band [*M* = 32.6, *SD* = 1.95 vs. *M* = 31.38, *SD* = 1.79; *t*_(36)_ = 3.31, *p* = 0.033] and in the gamma band [*M* = 38.23, *SD* = 1.51 vs. *M* = 37.29, *SD* = 1.82; *t*_(36)_ = 2.76, *p* = 0.01]. The global efficiency for nostalgic stimuli was significantly higher than that for non-nostalgic stimuli in the alpha band [*M* = 0.54, *SD* = 0.03 vs. *M* = 0.52, *SD* = 0.02; *t*_(36)_ = 3.31, *p* = 0.034] and in the gamma band [*M* = 0.64, *SD* = 0.02 vs. *M* = 0.62, *SD* = 0.03; *t*_(36)_ = 2.76, *p* = 0.037]. Similarly, local efficiency was significantly higher for nostalgic stimuli than non-nostalgic stimuli in the alpha band [*M* = 0.55, *SD* = 0.04 vs. *M* = 0.52, *SD* = 0.03; *t*_(36)_ = 3.3, *p* = 0.034] and in the gamma band [*M* = 0.64, *SD* = 0.03 vs. *M* = 0.62, *SD* = 0.03; *t*_(36)_ = 2.75, *p* = 0.036]. Furthermore, the eigenpath lengths for nostalgic stimuli were significantly shorter than those for non-nostalgic stimuli in the alpha band [*M* = 0.52, *SD* = 0.029 vs. *M* = 0.54, *SD* = 0.032; *t*_(36)_ = −3.32, *p* = 0.033] and in the gamma band [*M* = 0.62, *SD* = 0.03 vs. *M* = 0.64, *SD* = 0.02; *t*_(36)_ = −2.76, *p* = 0.01]. These results suggest that, in the alpha and gamma bands, nostalgic stimuli significantly enhanced the connection strength and global and local efficiency and shortened the feature path length of the whole-brain network. However, the effect of channel type is not significant on these indicators.

**Figure 5:**
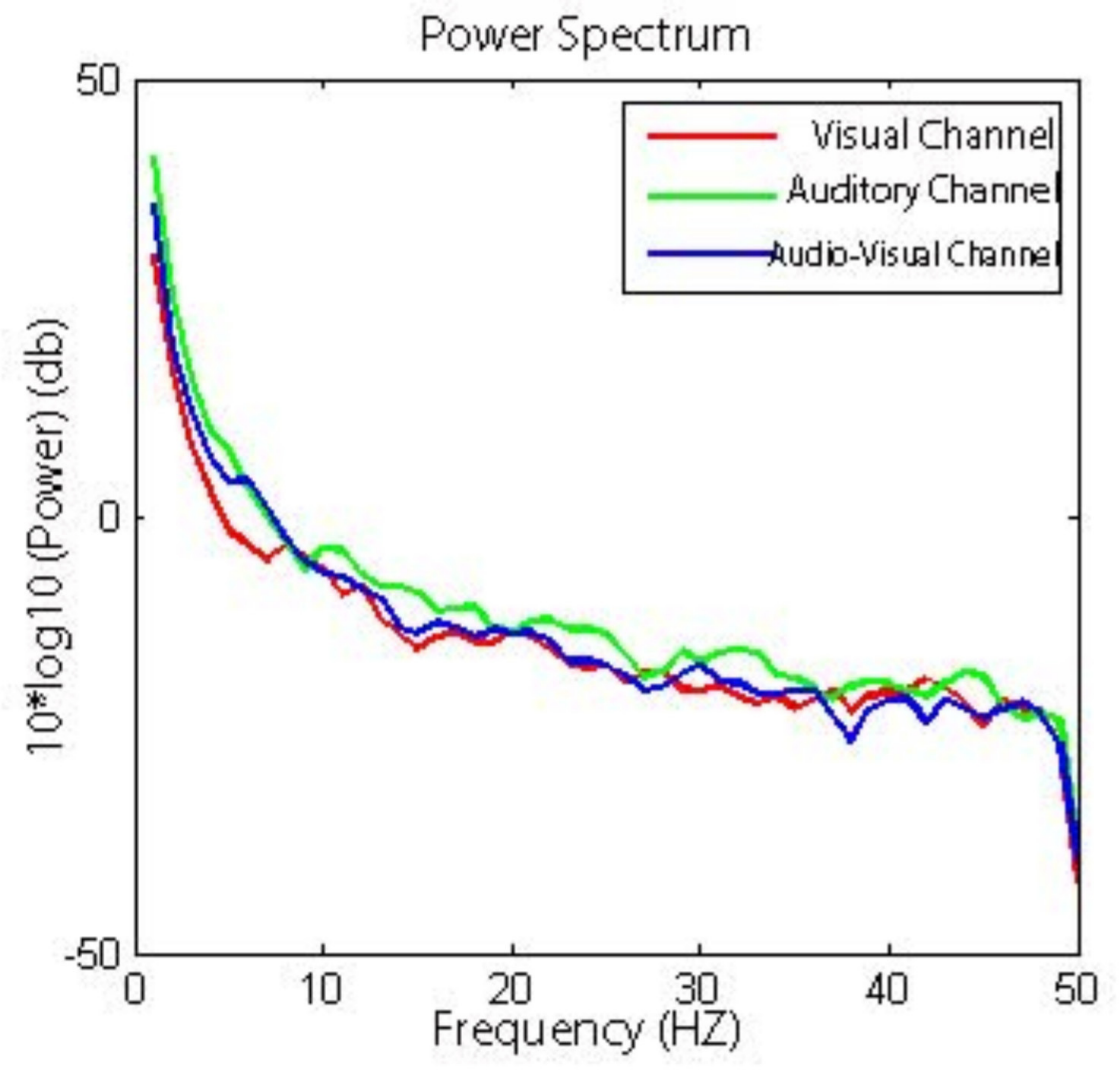
Power spectra of nostalgic music at different channels.

To compare neural activity across different sensory channels during the presence of nostalgic stimuli, frequency domain analysis was applied to the acquired EEG signals. The EEG time-series data were processed using the Fast Fourier Transform (FFT) to extract power features from the delta (1–4 Hz), theta (4–8 Hz), alpha (8–14 Hz), beta (14–31 Hz), and gamma (31–50 Hz) bands. Instead of calculating the power of individual frequencies, the aggregated power within each predefined frequency band was utilized to provide a comprehensive feature set. This method enables the assessment of neural responses across auditory, visual, and audiovisual conditions when participants listen to nostalgic material. The results of power changes of nostalgic stimuli through different sensory channels showed that neuronal activity in the auditory channel changed significantly more than that in the visual (*p* = 0.035) and audiovisual channels (*p* = 0.037; Figure 5).

**Figure 6:**
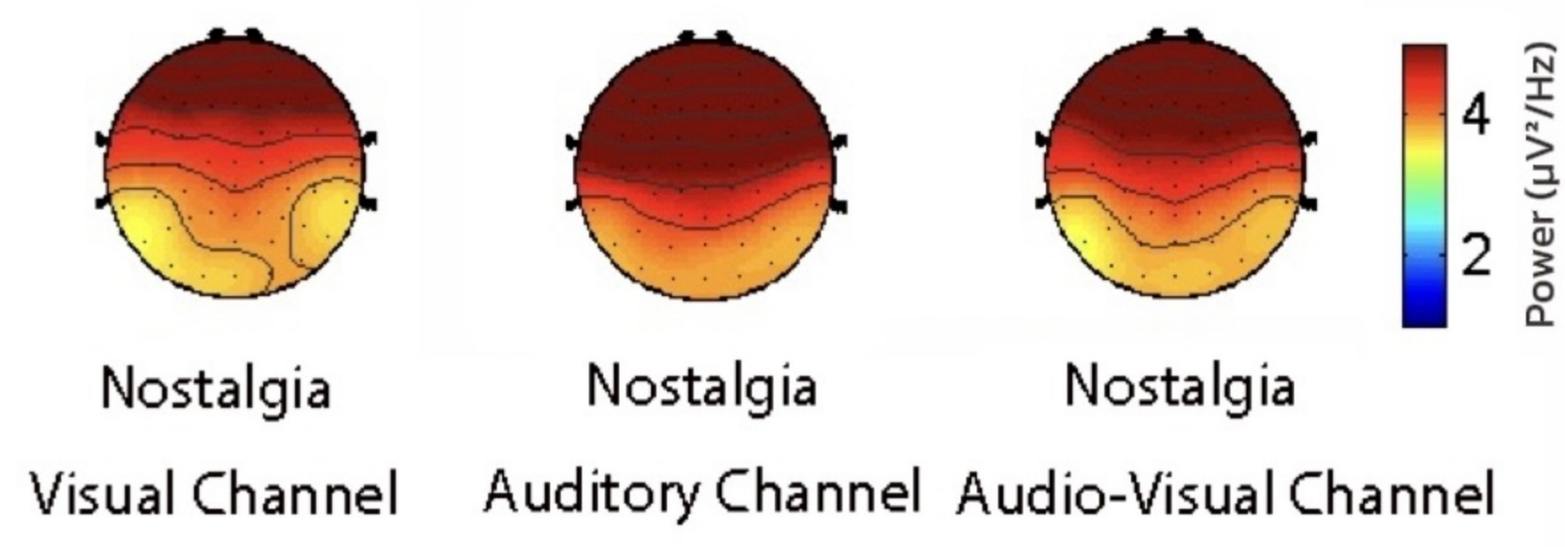
Comparison of power spectral density in the theta band.

**Figure 7:**
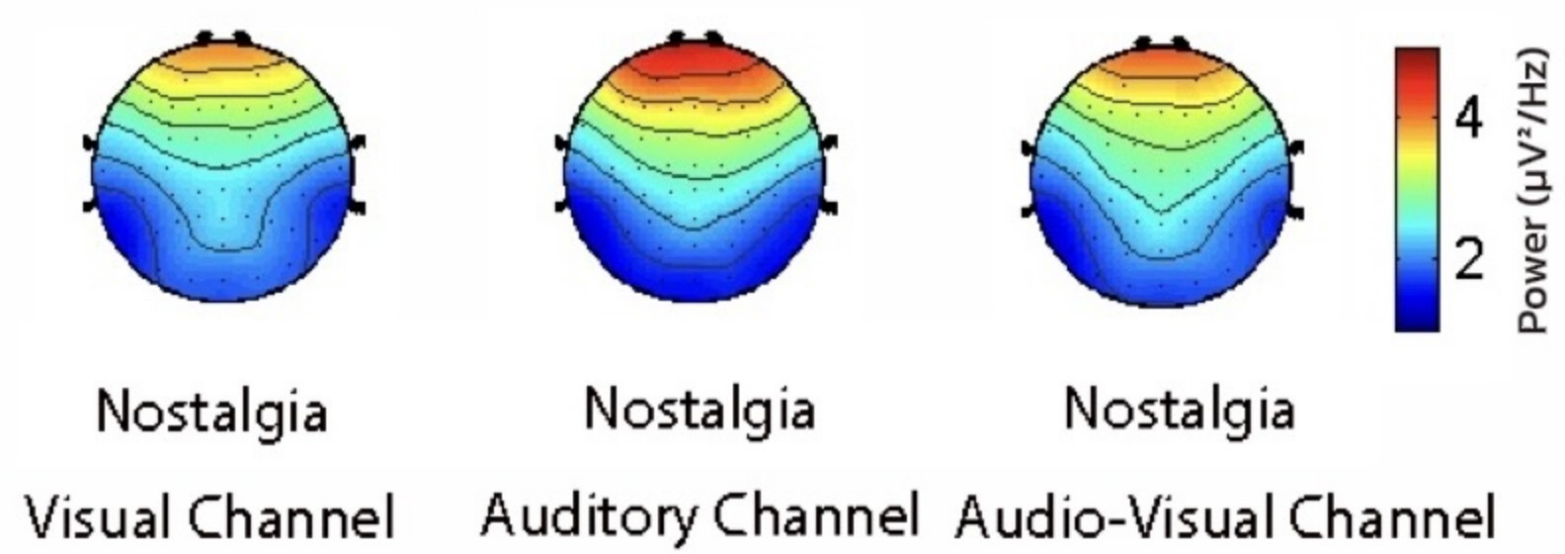
Comparison of power spectral density in the gamma band.

**Figure 8:**
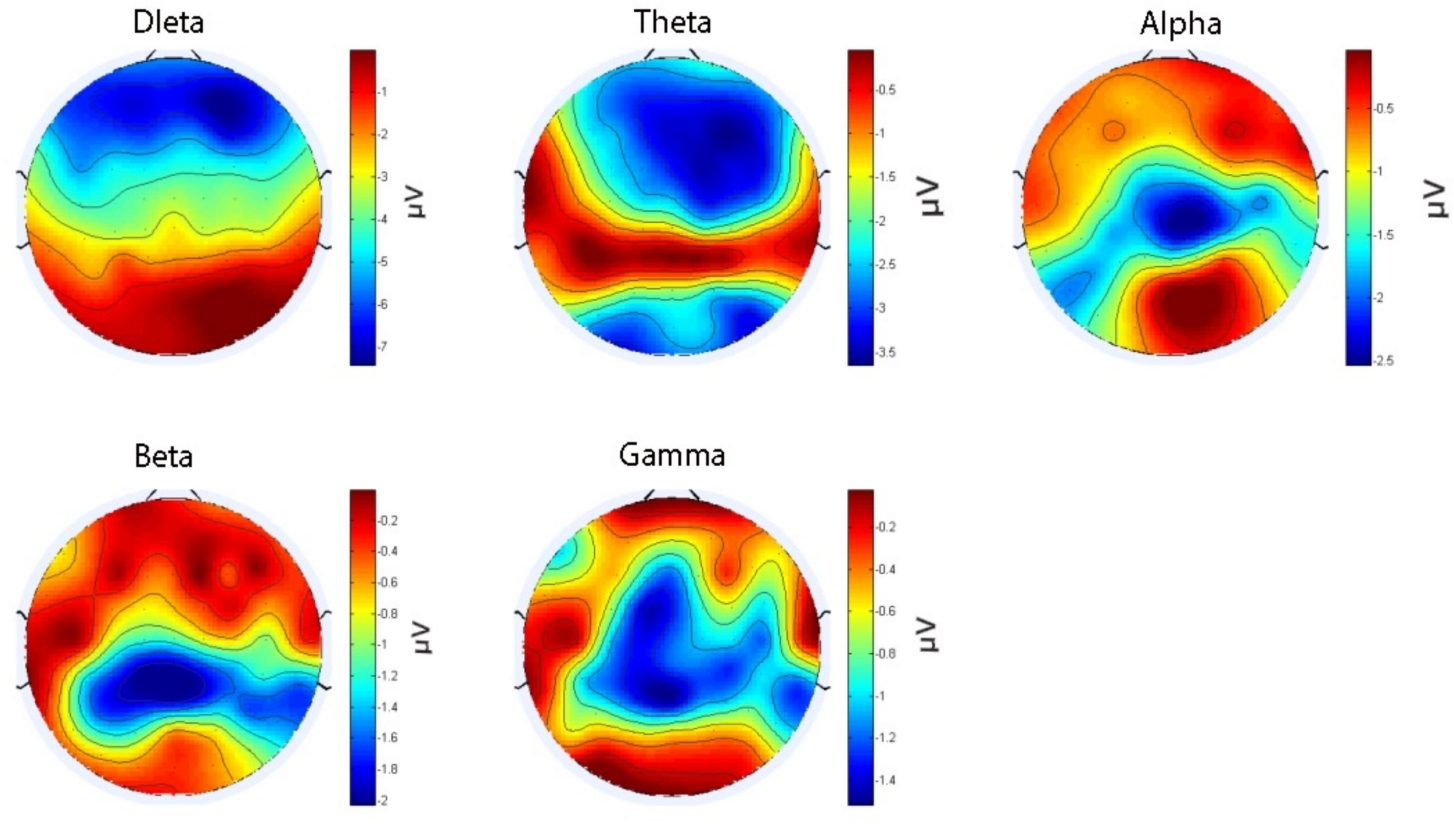
Comparison of nostalgic audiovisuals and nostalgic visuals.

**Figure 9:**
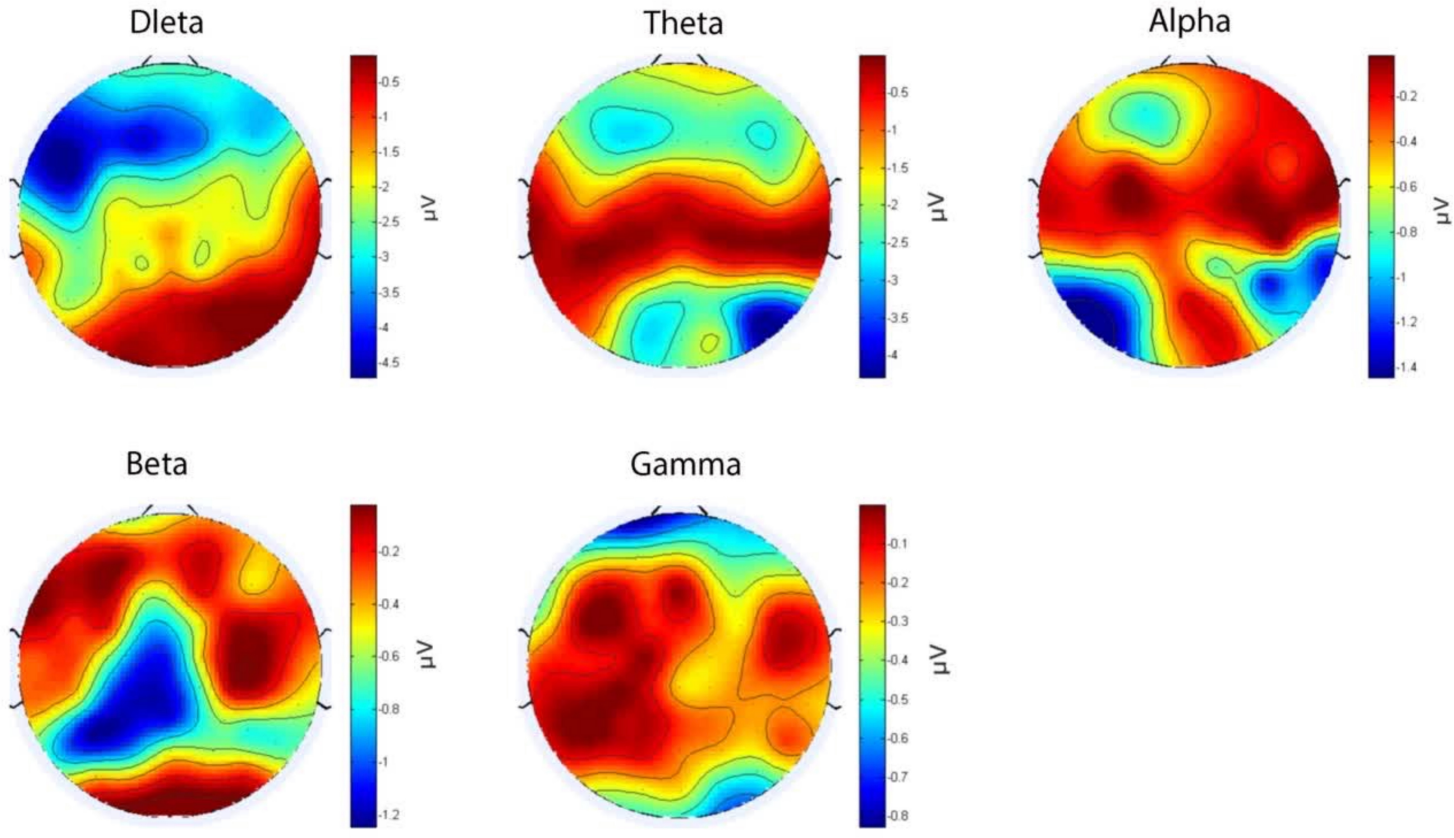
Comparison of nostalgic auditory and nostalgic audiovisual sensations.

To reveal the role of different sensory channels in the emotional responses triggered by nostalgic stimuli, EEG data from the visual, auditory, and audiovisual channels were statistically analyzed using repeated measures ANOVA. The results showed that the mean PSD (Power Spectral Density) (activity intensity) of the auditory channel in the prefrontal region was significantly higher than that of the visual (*p* = 0.0036) and audiovisual channels (*p* = 0.041) in the theta band (Figure 6). Moreover, the amplitude of the auditory channel in the central region was significantly higher than that of the visual channel (*p* = 0.013) in the gamma band (Figure 7). Further analysis showed that, in the alpha and beta frequency bands, the brainwave amplitudes evoked by auditory channels were significantly higher than those evoked by visual (*p* = 0.0023) and audiovisual channels (*p* = 0.032; Figure 8). In addition, in the alpha band, the wave amplitude intensity evoked by the audiovisual channel was significantly higher than that evoked by the visual channel (*p* = 0.033; Figure 9).

## 4 Discussion

The present study explored the behavioral and neural mechanisms of nostalgic stimuli across different sensory channels by analyzing emotional responses and brain network indicators. At the behavioral level, nostalgic stimuli elicited stronger state nostalgia, along with higher levels of pleasure, arousal, and dominance. At the neural level, analysis of WPLI-based brain network features in the time-frequency domain revealed that nostalgic stimuli enhanced connection strength, global efficiency, local efficiency, and reduced eigenpath length in the alpha and gamma frequency bands. Furthermore, in terms of brain activity within the time-frequency domain, the auditory channel evoked higher activity density in the theta and gamma bands and greater brainwave amplitudes in the alpha and theta bands. These findings highlight the neural and emotional processing advantages of nostalgic stimuli, particularly via the auditory channel.

### 4.1 Emotional responses of nostalgia from various modalities

In the current study, participants reported higher state nostalgia, pleasantness, arousal, and dominance when presented nostalgic stimuli compared to non-nostalgic stimuli. Research indicates that nostalgia engages brain regions such as the amygdala and hippocampus, emphasizing their roles in emotion regulation and overall well-being (Koelsch, 2018). This effect may arise from its ability to connect individuals to past experiences, fostering positive emotions. Induced nostalgic emotions positively correlated with perceived positive emotions, highlighting nostalgia as a key resource for enhancing emotions, social bonds, and self-perception. While most research defines nostalgia as a low-arousal emotion with positive affect, proximity orientation, and links to social connectedness and self-compassion (Van Tilburg et al., 2019), participants in this study reported higher arousal. Previous studies suggest nostalgia can trigger intense emotions, especially when recalling major life events or close relationships, leading to high emotional arousal (Koelsch, 2018). Furthermore, familiar old songs transported participants back to their youth, evoking excitement and heightened emotional engagement. As such, the use of upbeat, rhythmic music in both conditions may have influenced the results, as it typically evokes high arousal. Additionally, rhythmic patterns, lively melodies, and harmonious tones in nostalgic music synergistically evoked nostalgia and positive emotions, thereby intensifying arousal. In addition, the high arousal may tie to the reward mechanism in music listening, that is, the emotional reward not only enhances pleasure but also amplifies arousal, reinforcing nostalgia's role in emotion regulation (Sedikides et al., 2008).

An in-depth analysis revealed that the audiovisual channel evoked stronger nostalgia, highlighting the unique advantages of multisensory integration in enhancing emotional experiences. This result echoes the findings of Petrini et al. (2010) and emphasizes the importance of the emotional impact of multichannel information presented after integration during sensory integration. The findings during current study align with previous research suggesting that multisensory stimuli are more effective than unimodal stimuli in eliciting emotions (Barrett et al., 2010). Furthermore, the enhanced nostalgia associated with the audiovisual channel can be attributed to the integrated processing of visual and auditory cues, which creates a more immersive and emotionally engaging experience. This result extends earlier findings on sensory integration (Senkowski et al., 2008) by demonstrating the significant role of multisensory processing in nostalgia induction and emotional engagement.

### 4.2 Nostalgia and cognitive load

EEG results revealed significant increases in global and local efficiency, along with reduced eigenpath lengths, in participants' brain networks under nostalgic stimulation. Studies have shown the behavioral performance of the default mode network in the activated state (Greicius et al., 2003; Raichle et al., 2001), with a close correlation between the allocation of attentional resources (Koshino et al., 2014) and cognitive processing load. Weaker passive activation of the default mode network is associated with heavier cognitive load (Jenkins, 2019), requiring more attentional resources to engage in cognitive processing (Koshino et al., 2014). Our findings demonstrate that nostalgic materials significantly enhance the brain's processing capacity compared to non-nostalgic materials. This enhancement suggests a strong relationship between global efficiency and the increased strength of the brain network, as well as a positive cognitive state and emotional well-being during effective functioning. Specifically, nostalgia enhances the efficiency of the brain's information processing and task execution, leading to reduced cognitive load, decreased unnecessary energy expenditure, and facilitating faster and more accurate decision-making and cognitive activities. Moreover, the optimization of the network contributes to the effective regulation of emotional response, reduces anxiety and depressive symptoms, and promotes positive emotions.

### 4.3 Nostalgia and self-reflection

Nostalgia induced via the auditory channel resulted in greater brainwave amplitudes in the alpha bands. Studies indicate that nostalgic music with strong autobiographical associations significantly enhances alpha wave activity, driven by emotional arousal and relaxation. This is a biomarker of a relaxed state in the brain, especially when resting with the eyes closed or during light meditation. This state of relaxation helps individuals temporarily withdraw from the fast pace of daily life and promotes deeper thinking and introspection. Furthermore, alpha wave enhancement is not only related to emotion regulation but also plays a crucial role in creative thinking, which is an essential component of self-reflection (Fink and Benedek, 2014; Martindale and Mines, 1975). When alpha wave activity is enhanced, the brain is in a relaxed and open state, providing an ideal neurophysiological environment for deep thinking and self-reflection. Individuals in this state are more likely to have deeper emotions and memories that are difficult to retain in their busy daily lives. In addition, the enhancement of alpha waves helps extract emotionally rich memory fragments from long-term memory banks. Re-experiencing these memories not only triggers deep reflection on past experiences but also helps individuals construct and reshape their own identity perceptions and deepen their understanding of the connection between past experiences and their current state of self.

### 4.4 Nostalgia and autobiographical memory, emotion regulation and the brain's reward function

Brainwave amplitudes in the theta and gamma bands evoked by auditory nostalgia were significantly higher overall in the auditory channel compared to the visual channel and the audiovisual channel. Theta waves are typically observed during deep relaxation, meditation, and lighter phases of sleep (Kim et al., 2022). Theta wave activity is particularly important in emotional and memory processing. The enhancement of this low-frequency wave is closely related to the activation of deep personal memories, particularly when these memories are emotionally strongly charged. Nostalgic music triggers theta-wave enhancement by stimulating emotional memories associated with past experiences. These memories, which often contain important personal life events or moments related to intimate relationships, belong to participants' autobiographical memories. When theta wave activity is enhanced, it helps the brain extract these emotionally charged memories from long-term memory more efficiently while promoting the depth of the emotional experience, enabling individuals to mentally re-experience past emotional states.

Gamma waves are associated with higher cognitive functions, such as attentional focus, information processing, and perceptual integration (Strüber and Herrmann, 2022). The enhancement of gamma waves in the context of nostalgic music reflects the active state of the brain while processing complex emotional and cognitive tasks. When individuals listen to nostalgic music associated with autobiographical memories, it triggers emotionally related memories and stimulates deeper thinking about these memories. Enhanced gamma wave activity helps the brain integrate sensory information in the music with the emotional content of autobiographical memories and improves the ability to synchronize emotional and cognitive processing. Furthermore, when individuals begin to recall the autobiographical memories of past individuals, gamma wave activity can begin to increase significantly, which can aid in triggering and accurately reproducing autobiographical memories. Gamma waves are particularly useful in situations where complex and detailed information must be extracted from long-term memory. One study observed that in experiments involving music perception, gamma wave activity was significantly enhanced when participants listened to nostalgic music (Yang et al., 2020). This suggests that the brain's cognitive resources are highly mobilized to support the effective integration of emotional and cognitive information when processing complex emotions and memories triggered by nostalgic music. This indicates the involvement of relevant brain regions in autobiographic and emotional regulation parts of the brain when listening to nostalgic music.

The observed theta-band differences may also reflect the activation of reward mechanisms. Theta waves enhance activity in emotion-related brain regions, amplifying positive emotional responses driven by the reward system. This aligns with previous research indicating that nostalgia engages reward-related systems, such as the ventral striatum and medial prefrontal cortex, which are critical for reward processing and emotion regulation (Oba et al., 2016). Additionally, regions like the anterior cingulate cortex and orbitofrontal cortex are implicated in reward-related tasks, underscoring their role in processing nostalgic experiences. These neural circuits also link nostalgia to emotional resilience, promoting positive emotions while inhibiting negative ones (Speer et al., 2014). Our findings indicate that enhanced theta oscillations in anterior brain regions reflect a combination of preference processing and reward system activation. This mechanism further amplifies positive emotional responses.

In summary, nostalgic music effectively regulates emotions and enhances subjective well-being from both neuroscientific and behavioral perspectives. Electroencephalography (EEG) studies have revealed that nostalgic music significantly enhances the brain network activity in the Alpha, Theta, and Gamma bands compared to non-nostalgic music, suggesting that nostalgic music promotes the brain's processing ability in emotional memory extraction, cognitive load reduction, and deepening of emotional experiences. In addition, the intensity of nostalgia induced by a single auditory channel was higher than that induced by multiple channels, suggesting that the complexity of multichannel information processing may interfere with the emotional experience of nostalgic music. These findings underscore the positive role of nostalgic music in emotion regulation and elucidate its underlying neural mechanisms. This study provides experimental evidence for the effectiveness of nostalgic music in psychological healing and emotional interventions. Our findings not only reveal the emotion regulation mechanism of nostalgic music from the perspective of brain neuroscience but also provide references for its practical application in psychotherapy and emotional intervention.

While the present study provides significant insights into the neural mechanisms of nostalgia and demonstrates the effectiveness of nostalgic music in emotion regulation and enhancing subjective well-being, several limitations should be acknowledged. Firstly, the reliance on self-report methods to assess emotional experiences introduces potential variability due to individual psychological states and cognitive biases, which may affect the accuracy of the results. Future research could benefit from more objective measurement techniques, such as pretest-posttest designs and functional magnetic resonance imaging (fMRI). Additionally, the study's focus on university students offers a controlled and homogeneous sample but limits the generalizability of the findings to broader populations. Future studies should include a more diverse participant pool, encompassing older adults and individuals from various backgrounds, to enhance the applicability of the conclusions. Moreover, incorporating variables such as personality traits, character, and upbringing could provide deeper insights into the neural underpinnings of nostalgia. Despite these limitations, this study makes important contributions by demonstrating the emotional and neural effects of nostalgic stimuli through different sensory channels. It highlights the potential of nostalgia as an emotional resource, offering valuable insights into its role in enhancing well-being and cognitive processing. Addressing these limitations in subsequent studies will enable a more comprehensive and generalizable understanding of nostalgia's neural mechanisms and its therapeutic potential in psychological healing and emotional interventions.

## Data Availability

The raw data supporting the conclusions of this article will be made available by the authors, without undue reservation.
