# Supplementary material for: Harmonizing the past: EEG-based brain network unveil modality-specific mechanisms of nostalgia
Source: Front Psychol. 2025 Jan 22;16:1517449. doi: 10.3389/fpsyg.2025.1517449 (PMC11794493; doi:10.3389/fpsyg.2025.1517449)
Supplement: Supplementary file 1 [file Table_1.docx]

Supplementary Material：

TableS1 Nostalgic Songs for College Students Screening Results

| Song Title | Sample Size | Nostalgia Average | Pleasure Average | Familiarity Average |
| --- | --- | --- | --- | --- |
| Big Windmill (1997) | 257 | 4.607 | 4.319 | 4.619 |
| Little Apple (2014) | 257 | 4.584 | 4.307 | 4.572 |
| SpongeBob (2000) | 257 | 4.58 | 4.529 | 4.626 |
| Let's Row the Boat Gently (1955) | 257 | 4.572 | 4.16 | 4.49 |
| Invisible Wings (2006) | 257 | 4.56 | 4.183 | 4.401 |
| Happy Flying Forward (2013) | 257 | 4.549 | 4.42 | 4.553 |
| Where Did the Time Go (2012) | 257 | 4.521 | 3.794 | 4.475 |
| Road to Ordinary (2014) | 257 | 4.514 | 4.257 | 4.498 |
| Against the War (2012) | 257 | 4.502 | 4.35 | 4.588 |
| Blue and White Porcelain (2007) | 257 | 4.494 | 4.307 | 4.475 |
| Sunny Boy Sunny Girl (2005) | 257 | 4.486 | 4.436 | 4.475 |
| Big Ears Totu (2004) | 257 | 4.463 | 4.315 | 4.584 |
| Welcome to Beijing (2008) | 257 | 4.459 | 4.202 | 4.327 |
| Preference (2009) | 257 | 4.44 | 4.121 | 4.409 |
| My Motherland (1956) | 257 | 4.436 | 4.132 | 4.257 |
| Light Seeker (2017) | 257 | 4.412 | 4.132 | 4.393 |
| Just Met You (2016) | 257 | 4.397 | 4.163 | 4.444 |
| Actor (2015) | 257 | 4.389 | 4.039 | 4.432 |
| Pleasant Goat and Big Big Wolf (2005) | 257 | 4.374 | 4.043 | 4.377 |
| See You Again (2015) | 257 | 4.37 | 4.121 | 4.381 |
| Love Balloon (2016) | 257 | 4.346 | 4.35 | 4.444 |
| Glorious Years (1990) | 257 | 4.342 | 3.743 | 4.409 |
| Chengdu (2016) | 257 | 4.3 | 4.027 | 4.28 |
| Boundless Oceans, Vast Skies(1993) | 257 | 4.237 | 3.969 | 4.261 |
| Fairy Tale (2005) | 257 | 4.233 | 3.817 | 4.047 |
| Cool Down (2017) | 257 | 4.226 | 3.848 | 4.374 |
| Miracle Again (2012) | 257 | 4.218 | 4.047 | 4.093 |
| Little Luck (2016) | 257 | 4.214 | 3.914 | 4.183 |
| Intermission Exercise Music | 257 | 4.202 | 3.113 | 4.319 |
